# Toxic and Sublethal Effects of Commonly Used Pesticides on *Neoseiulus barkeri* (Acari: Phytoseiidae)

**DOI:** 10.3390/insects17090891

**Published:** 2026-08-25

**Authors:** Yu-Fei Chen, Zhen Wu, Yu-Chao Shen, Teng-Ye Yin, Si-Chen Li, Liu Liu, Qi Pan, Wei Zhang, Lin Cong, Wan-Fu Yao, Shi-Jiang Yu, Chun Ran

**Affiliations:** 1 Citrus Research Institute, Southwest University, National Engineering Research Center for Citrus, Chongqing 400712, China; 2 Yuxi Academy of Agricultural Sciences, Yuxi 653100, China

**Keywords:** integrated pest management, non-target effects, population life table, toxicity bioassay

## Abstract

*Neoseiulus barkeri* is an important predatory mite used for biological control in citrus orchards in China. Systematic studies on the toxicity of commonly used pesticides to predatory mites are rarely reported. In this study, we developed a “four-well volcanic dish” bioassay system to determine the acute contact toxicity of 33 pesticides registered in citrus, and further evaluated the sublethal effects of chlorpyrifos, lambda-cyhalothrin, and chlorfenapyr at LC_10_ and LC_20_ on the development and reproduction of *N. barkeri*. Amitraz was the most toxic compound, with an LC_50_ of 55.92 mg/L. Chlorpyrifos, chlorfenapyr, lambda-cyhalothrin, glufosinate-ammonium, and beta-cyfluthrin also showed relatively high toxicity, with LC_50_ values of 100–400 mg/L, whereas many other pesticides were much less toxic, with LC_50_ values above 1000 mg/L. LC_20_ exposure to chlorpyrifos, lambda-cyhalothrin, or chlorfenapyr significantly prolonged development, reduced fecundity, and shortened female longevity. LC_20_ treatments also decreased the net reproductive rate (*R*_0_) and intrinsic rate of increase (*r_m_*), indicating reduced population growth potential. This study clarified the toxic effects of various pesticides on predatory mites and further elucidated the sublethal effects of three broad-spectrum pesticides on these mites. These findings provide a theoretical basis for coordinating chemical and biological control in citrus IPM programs.

## Simple Summary

The predatory mite *Neoseiulus barkeri* is a widely used beneficial natural enemy in Chinese citrus orchards, as it preys on small agricultural pests such as spider mites and thrips to reduce crop damage. While chemical pesticides are the primary tool for pest control in citrus production, they often harm these beneficial mites and undermine biological control efforts, and the specific impacts of most common citrus pesticides on this predatory mite have not been systematically assessed. In this study, we tested the acute toxicity of 33 commonly used citrus pesticides in commercial formulations to *N. barkeri* with a customized bioassay device. These pesticides can be divided into four major categories based on their targets and modes of action (acaricides, insecticides, fungicides and herbicides). We further examined how low residual levels of three widespread insecticides affect the mite’s growth, reproduction, and offspring. We found striking differences in pesticide safety: some broad-spectrum insecticides and acaricides were highly toxic, whereas most fungicides and selective insecticides posed very low risk. Low sublethal doses of the three tested insecticides slowed development, reduced fecundity, shortened adult lifespan, weakened population growth, and caused negative transgenerational effects. These results provide guidance for selecting pesticides compatible with beneficial mites, supporting balanced and more sustainable pest management in citrus farming.

## 1. Introduction

*Neoseiulus barkeri* Hughes, 1948 (Acari: Phytoseiidae) is a predatory mite widely distributed across China, with a confirmed worldwide range covering Africa, the Americas, Europe, Asia and Oceania [1,2,3]. This mite has a broad host range including citrus, deciduous fruit trees, vegetables, field crops and ornamental plants, and also occurs commonly in stored product environments. It preys effectively on a range of small agricultural pests and mites, including spider mites, thrips, whiteflies, and plant-parasitic nematodes [4,5,6]. Owing to its short developmental duration, high reproductive capacity, and strong adaptability, *N. barkeri* is widely recognized as an effective biocontrol agent for the biological control [7].

Chemical control remains the dominant pest management strategy in commercial agricultural production. However, the application of pesticides inevitably poses significant non-target risks to *N. barkeri*, which often leads to severe conflicts between chemical pesticide application and the field release of *N. barkeri*—especially when the susceptibility of this predatory mite to different pesticides remains poorly understood. Therefore, screening pesticides with low toxicity to *N. barkeri* is a feasible and practical strategy to coordinate chemical and biological control, by mitigating the adverse non-target effects of chemical agents on this key natural enemy [8].

In China, *N. barkeri* has become one of the most commercially produced predatory mite species [9], and is extensively applied for the management of *Panonychus citri*—a key pest that poses a severe threat to citrus yield and fruit quality [10]. However, the toxicity of pesticides commonly used in citrus orchards to *N. barkeri* under commercial field conditions remains poorly characterized. Pesticides routinely applied in citrus orchards are mainly divided into four major classes: insecticides, acaricides, fungicides, and herbicides. Broad-spectrum insecticides, including organophosphates and pyrethroids, are widely documented to exert adverse effects on phytoseiid predatory mites, and their over-application can significantly reduce the pest control efficacy of natural enemies [11]. For instance, chlorpyrifos has been confirmed to be toxic to a wide range of predatory natural enemies [12,13], with a mortality rate exceeding 80% in *N. barkeri* [14]. Among acaricides, pyridaben exhibits relatively high toxicity to the nymphal stages of *N. barkeri* [15]. In contrast, the non-target effects of most fungicides and herbicides, such as glufosinate-ammonium, on *N. barkeri* remain largely unknown [16]. Therefore, it is necessary to comprehensively evaluate the toxicity of commonly used pesticides in citrus orchards to *N. barkeri*.

In addition to direct toxic effects, pesticide residues under field conditions can also exert sublethal effects on *N. barkeri* [17], including impairment of its developmental process and physiological functions, as well as transgenerational effects on its population fitness [18]. For instance, residual exposure to abamectin and chlorfenapyr has been documented to adversely affect the reproduction and immature stage development of *N. barkeri* [19,20]. Sublethal concentrations of pyridaben can reduce the fecundity of female *N. barkeri.* and decrease egg survival rate [21]. Organophosphates and pyrethroids not only exhibit high toxicity to *N. barkeri.*, but also trigger remarkable sublethal effects. Specifically, sublethal concentrations of chlorpyrifos have been reported to markedly reduce both the fecundity and adult longevity of female *N. barkeri* [22]. Accordingly, clarifying the impacts of sublethal doses of commonly used pesticides on the growth, development, and reproduction of *N. barkeri* is critical to achieving the synergistic application of this key predatory mite and pesticides in agricultural pest management.

Despite increasing interest in chemical toxicity to predatory mite, a scarcity of systematic analysis can be observed. In this study, we systematically determined the toxicity of 33 pesticides commonly used in citrus orchards, and further evaluated the sublethal effects of the three broad-spectrum insecticides on the development and reproduction of *N. barkeri*. The findings of this study provide a critical theoretical basis for improving the compatibility between chemical and biological control strategies, and facilitate the effective incorporation of *N. barkeri* into integrated pest management (IPM) systems for sustainable citrus pest management [23].

## 2. Materials and Methods

### 2.1. Pesticides and Mites

The tested *N. barkeri* population was originally collected from citrus orchards in 2010, and has been continuously reared in a dedicated indoor facility without any exposure to pesticides since then. The rearing protocol for *N. barkeri* was as follows: *Aleuroglyphus ovatus* (prey for *N. barkeri*) was reared on sterilized wheat bran in sealed plastic rearing containers (length × width × height, L × W × H: 35 cm × 25 cm × 20 cm). Once the prey population had expanded to a sufficient density, a standardized number of *N. barkeri* adults were introduced into the containers for mass rearing. Each rearing container was placed in an individual water-filled plastic tray, with the outer rim of the container coated with insect barrier glue to preclude mite escape. All rearing units were maintained in a climate-controlled incubator at 26 °C, 70% relative humidity (RH), and a 16 h light: 8 h dark (16L:8D) photoperiod. The population abundance of both prey mites and *N. barkeri* was monitored daily, and the rearing procedure was repeated when the prey supply was depleted.

Thirty-three tested pesticides were used in this study, which were categorized into four major classes: acaricides, insecticides, fungicides, and herbicides. To ensure the test results are consistent with actual field application scenarios in citrus production, all bioassays were performed using commercial formulations of the tested pesticides, rather than technical grade active ingredients. Detailed information on these pesticides was listed below:

Acaricides: Amitraz (200 g/L EC, Arysta LifeScience Corporation, Tokyo, Japan), Pyridaben (15% EC, Yancheng Shuangning Agrochemical Co., Ltd., Yancheng, China), Propargite (13% EW, Nihon Nohyaku Co., Ltd., Tokyo, Japan), Spirodiclofen (240 g/L SC, Mertech USA, Inc., Houston, TX, USA), Azocyclotin (20% SC, Shenzhen Noposion Crop Science Co., Ltd., Shenzhen, China), Fenpyroximate (5% SC, Shanghai Huiguang Environmental Technology Co., Ltd., Shanghai, China), Bifenazate (43% SC, Zhejiang Xinan Chemical Industrial Group Co., Ltd., Hangzhou, China), Fenbutatin oxide (20% SC, Tianjin Hanbang Plant Protectant Co., Ltd., Tianjin, China), Tolfenpyrad (15% SC, Shaanxi Pucheng Meierguo Agrochemical Co., Ltd., Weinan, China).

Insecticides: Chlorpyrifos (480 g/L EC, Nufarm Australia Ltd., Melbourne, VIC, Australia), Chlorfenapyr (10% SC, Liuzhou Hui Nong Chemical Co., Ltd., Liuzhou, China), Beta-cyfluthrin (2.5% EW, Liuzhou Hui Nong Chemical Co., Ltd., Liuzhou, China), Lambda-cyhalothrin (25 g/L EC, Shandong Wokang Biotechnology Co., Ltd., Weifang, China), Flupyradifurone (17% SL, Bayer AG, Leverkusen, Germany), Thiamethoxam (25% WDG, Syngenta Crop Protection AG, Basel, Switzerland), Imidacloprid (20% SL, Shanghai Dongfeng Pesticide Factory Co., Ltd., Shanghai, China), Nitenpyram (10% AS, Guangdong New Vision Bioengineering Co., Ltd., Yangjiang, China), Pyriproxyfen (100 g/L EC, Shanghai Shennong Biochemical Products Co., Ltd., Shanghai, China), Abamectin (3.2% EC, Qingdao Zhongda Agricultural Technology Co., Ltd., Qingdao, China), Buprofezin (25% WP, Nihon Nohyaku Co., Ltd., Tokyo, Japan), Spirotetramat (22.4 g/L SC, Bayer AG, Leverkusen, Germany), Chlorantraniliprole (200 g/L SC, FMC Corporation, Philadelphia, PA, USA).

Fungicides: Mancozeb (80% WP, Sinon USA, Inc., Lafayette, CA, USA); Fluazinam (500 g/L SC, Jiangsu Dongbao Agrochemical Co., Ltd., Yangzhou, China); Captan (80% WDG, East Coast Resources Australia Pty Ltd., Australia); Pyraclostrobin (30% SC, Shanghai Yuelian Biotechnology Co., Ltd., Shanghai, China); Prochloraz (250 g/L EC, FMC Corporation, Philadelphia, PA, USA); Copper hydroxide (77% WP, Nufarm Limited, Melbourne, VIC, Australia); Thiophanate-methyl (70% WP, Sankyo Co., Ltd., Tokyo, Japan); Difenoconazole (10% WDG, Syngenta Crop Protection AG, Basel, Switzerland); *Bacillus subtilis* (10 billion CFU/g, WP, BioWorks, Victor, NY, Inc., USA).

Herbicides: Glufosinate-ammonium (200 g/L AS, Mertech USA, Inc., Fulshear, TX, USA); Glyphosate (41% AS, Mertech USA, Inc., Fulshear, TX, USA).

### 2.2. Toxicity Determination of 33 Pesticides to N. barkeri

**Figure 1:**
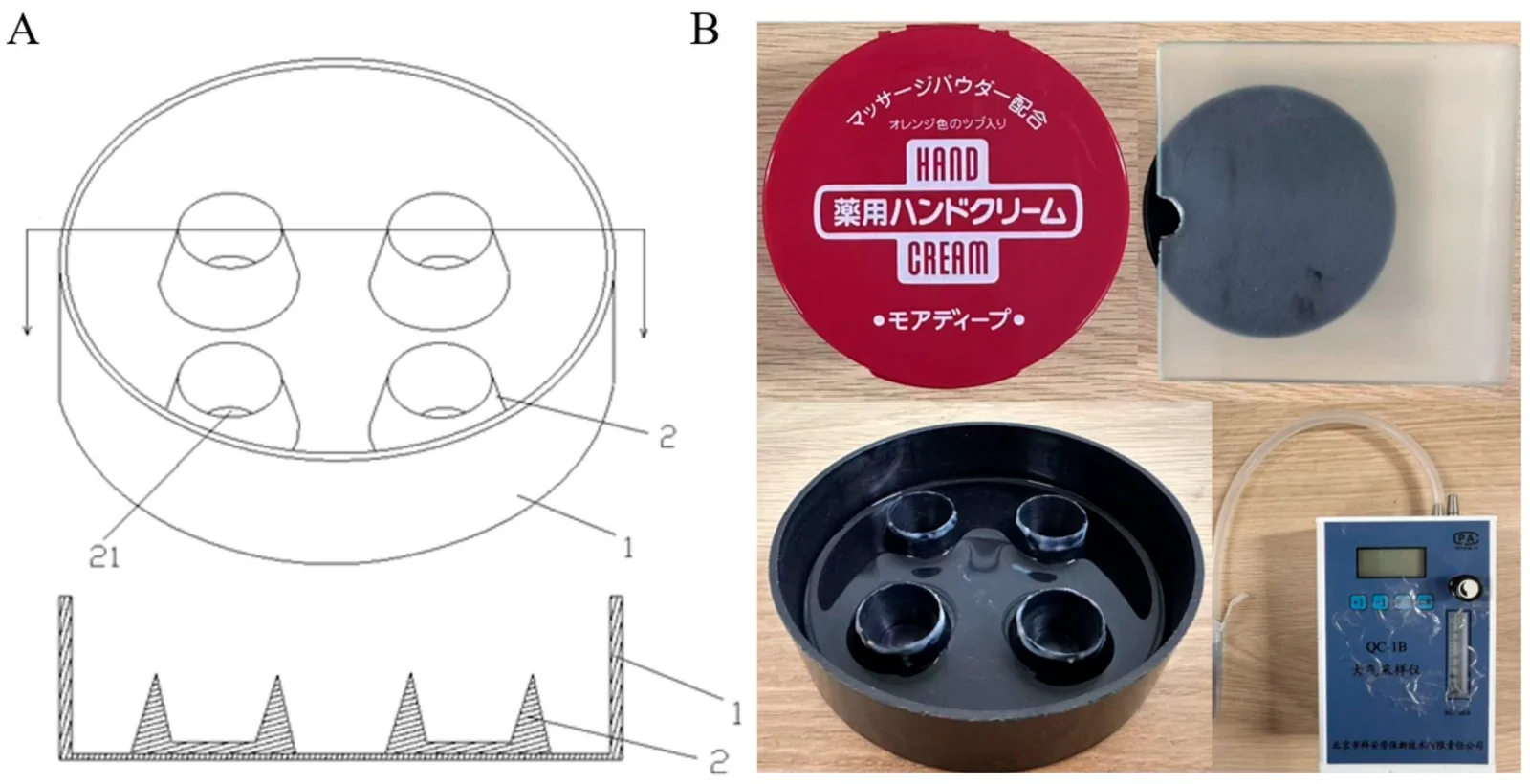
(**A**) Bioassay device for *Neoseiulus barkeri*. This device, 7 cm in diameter and 2 cm in height, contains four wells at distinct positions. Each well’s inner wall cross-section forms an inverted trapezoid—wider at the top and narrower at the bottom—with an inner diameter of 1 cm (bottom), 1.5 cm (top), and a depth of 1 cm. The outer wall cross-section of each well is a regular trapezoid, narrower at the top and wider at the bottom. This design facilitates the uniform, vertical settlement of pesticide spray on both the inner and outer well walls during spray tower applications, guaranteeing consistent coverage. It also eliminates blind spots for mite observation under a stereomicroscope, supporting accurate counting. Moreover, the area surrounding the wells can hold water to sustain the humidity required for mite survival and prevent their escape. (**B**) Shows the collection device used for toxicity bioassays and the anti-escape setup for the mite population. Non-English labels: The Japanese texts describe a medicated hand cream (Moadeep series) containing massage powder and orange-colored particles. The Chinese label on the instrument denotes “air sampler”, and the manufacturer name below is Beijing Ke’an Labor Protection New Technology Co., Ltd.

Owing to the high crawling speed and strong obstacle-crossing ability of *N. barkeri* [24], we optimized the standard bioassay protocol and developed a specialized testing device for this predatory mite, which we named the “four-well volcanic dish” (Figure 1). Following custom mold fabrication, we manufactured this device via a standardized one-step injection molding process using a precision injection molding machine.

The toxicity bioassay was conducted as follows (Figure 1B): A standardized volume of purified water was added to the area surrounding the four wells at the base of the “volcanic dish”, and urea hand cream (Shiseido, Tokyo, Japan; Catalog No.: 49325263) was applied to the inner upper walls and top edges of the four wells to prevent test mites from escaping. Using a handheld aspirator (Model QC-1B, Beijing Kean Instruments Co., Ltd., Beijing, China), 10–20 healthy adults of *N. barkeri* were transferred into each well for the bioassay. One to two pieces of wheat bran and a small number of prey mites were added to each well to facilitate the climbing and predation of *N. barkeri*, while ensuring that these additions did not interfere with the counting of test mites. The initial total number of live *N. barkeri* in each well was recorded immediately after inoculation.

All tested pesticides were registered commercial formulations and diluted with purified water. Based on preliminary range-finding tests, 5–7 serial concentrations were set for each pesticide to achieve a 10–90% corrected mortality range for subsequent probit regression analysis. For each treatment, 2 mL of test solution was uniformly sprayed onto the inner surface of the “four-well volcanic dish” using a standard Potter precision spray tower (Burkard Manufacturing Co Ltd, Uxbridge, United Kingdom). Each device represented one replicate, and the experiment was independently repeated at least three times. The control group was sprayed with an equal volume of purified water following the same procedure.

After the sprayed pesticide solution had settled evenly on the inner surface of the wells, each device was covered with a glass plate, with narrow air-permeable gaps reserved between the plate and the device to maintain stable internal humidity. All devices were then transferred to a climate-controlled incubator set at 27 °C, with a 16L:8D photoperiod. Mite mortality was assessed at 24 h post-treatment; mites were considered dead if no limb movement was observed after gentle probing [22]. The bioassay was valid only if control mortality was ≤10%, and treatment mortality was corrected using Abbott’s formula. Toxicity data were analyzed by probit regression using PoloPlus software (Version 2.0, LeOra Software, Petaluma, CA, USA).

### 2.3. Sublethal Effects of Chlorpyrifos, Lambda-Cyhalothrin, and Chlorfenapyr on the Development of the Parental Generation (F_0_) of N. barkeri

Twenty newly laid eggs of *N. barkeri* were transferred to a “four-well volcanic dish” device, then sprayed with 2 mL of test pesticide solution using a Potter precision spray tower. Three insecticides (chlorpyrifos, lambda-cyhalothrin, chlorfenapyr) were tested at their respective sublethal concentrations (LC_10_ and LC_20_), corresponding to the 24 h acute toxicity values for *N. barkeri* female adults determined in this study. After air-drying, devices were transferred to a climate-controlled incubator under the same rearing conditions as the stock colony (27 °C, 70% RH, 16L:8D photoperiod). The completion of spraying and air-drying was set as the observation starting point (t = 0). The control group was treated with an equal volume of purified water following the same protocol. Each treatment was independently replicated three times. Upon hatching, each *N. barkeri* larva was transferred to an individual unit for single-mite rearing. Larvae of *A. ovatus* were supplied daily as prey. Observations were conducted every 6–10 h to record key developmental time points. The duration of each developmental stage was calculated after all mites had reached the adult stage or died.

To facilitate presentation in subsequent tables and results, chlorpyrifos, lambda-cyhalothrin, and chlorfenapyr were designated as Group A, Group B, and Group C, respectively. The corresponding sublethal concentrations for female adults are abbreviated as Aa-LC_10_/Aa-LC_20_, Ba-LC_10_/Ba-LC_20_, and Ca-LC_10_/Ca-LC_20_, where the lowercase letter “a” denotes the adult stage used for acute toxicity bioassays. When replaced with the lowercase letter “e”, the abbreviations refer to sublethal concentrations determined for the egg stage of *N. barkeri*.

### 2.4. Sublethal Effects of Chlorpyrifos, Lambda-Cyhalothrin, and Chlorfenapyr on the Reproduction of Female Adults (F_0_) and the Development of F_1_ Generation

A sufficient number of *N. barkeri* eggs were collected and reared to adulthood to obtain virgin adult females for the experiment. Each virgin female was treated individually in a “four-well volcanic dish” via spray application with sublethal concentrations (LC_10_ and LC_20_) of chlorpyrifos, lambda-cyhalothrin, and chlorfenapyr under single-rearing conditions. The control group was treated with an equal volume of purified water following the same protocol. Twenty-four hours later, each surviving virgin female (F_0_ generation) was transferred to an individual well of a new “four-well volcanic dish” for individual rearing One newly emerged, unmated healthy adult male from the same stock colony was added to each well, and successful mating was confirmed after the male’s introduction. The number of eggs laid was recorded every 6–10 h, with eggs removed immediately after counting, until all F_0_ females died naturally.

To evaluate the transgenerational sublethal effects of the tested insecticides, forty eggs of *N. barkeri* (≤6 h post-oviposition, F_1_ generation) were randomly selected from the eggs laid by F_0_ females in each treatment group. Each egg was transferred individually to a separate well of a clean, pesticide-free “four-well volcanic dish” device (1 egg per well) to avoid cannibalism during development. The completion of egg transfer was designated as the starting point of developmental observation for the F_1_ generation (t = 0). All devices were maintained under the same standard rearing conditions as the stock colony, with sufficient prey supplied daily after hatching. The timing of key developmental events of F_1_ mites was recorded every 6–10 h until all individuals had either completed development to adulthood or died.

### 2.5. Construction of the Experimental Population Life Table

Age-specific life table parameters were calculated for each treatment group according to the method described by Birch (1948) [25]. The key parameters and their calculation formulas are defined below:

Net Reproductive Rate (*R*_0_): the mean number of female offspring produced by a single female mite throughout her lifespan, which represents the net multiplication rate of a population over one generation. It is calculated as:

$$R_{0}=\sum_{x=0}^{n}l_{x}\cdot m_{x}$$

Intrinsic Rate of Increase (*r_m_*): the maximum instantaneous growth rate of a population under stable age structure and unlimited ideal environmental conditions.

To ensure mathematical accuracy and avoid approximation errors for species with overlapping generations, the *r_m_* was estimated via the iterative bisection method based on the Euler–Lotka equation. To match the time window of our daily observation and eliminate systematic deviation caused by time mismatch, the discrete form of the equation was set as:

$$\sum_{x=0}^{n}e^{-r_{m}\cdot \left(x+1\right)}\cdot l_{x}\cdot m_{x}=1$$

Finite Rate of Increase (*λ*): the proportional change in population size per unit time (1 d), calculated as:

$$\lambda =e^{r_{m}}$$

Population Doubling Time (*D_t_*): the time required for a population to double in size under the given conditions, calculated as:

$$D_{t}=\frac{ln2}{r_{m}}$$

Mean Generation Time (*T*): the average time required for a female cohort to complete one generation, calculated as:

$$T=\frac{\sum_{x=0}^{n}(x+1)\cdot l_{x}\cdot m_{x}}{R_{0}}$$

In the above formulas: *x* is the age of female mites in days; *l_x_* is the age-specific survival rate, i.e., the probability that a newly hatched female mite survives to age *x*;

*m_x_* is the age-specific fecundity rate, i.e., the mean number of female eggs produced per surviving female mite at age *x*; *e* is the base of the natural logarithm. The standard errors of all life table parameters were estimated via the bootstrap method with 100,000 resamplings.

## 3. Results

### 3.1. Acute Toxicity of 33 Tested Pesticides to N. barkeri

The acute contact toxicity of 33 commonly used pesticides in citrus orchards, categorized into four classes (9 acaricides, 13 insecticides, 9 fungicides, and 2 herbicides), against *N. barkeri* was determined in this study (Table 1). The tested pesticides exhibited extremely significant divergence in toxicity to the predatory mite, with the median lethal concentration (LC_50_) values ranging from 55.92 mg a.i./L to over 40,000 mg a.i./L (a more than 700-fold span). The goodness-of-fit of all probit regression models was validated by the chi-square test, confirming the reliability of the toxicity estimation.

For different pesticide categories: amitraz showed the highest toxicity among acaricides, while bifenazate and fenbutatin oxide presented extremely high safety to *N. barkeri*. Insecticides had the most dramatic variation in toxicity: broad-spectrum insecticides (organophosphates and pyrethroids) exerted extremely high acute toxicity, whereas selective insecticides (e.g., thiamethoxam, spirotetramat) showed no significant acute lethal effect on the predatory mite. Most tested fungicides had an excellent safety profile, with only prochloraz and mancozeb showing low-level toxicity, and the rest had no notable acute lethal effect. For herbicides, glufosinate-ammonium exhibited moderate toxicity, while glyphosate had no obvious acute toxicity to *N. barkeri*.

### 3.2. Toxicity of Chlorpyrifos, Lambda-Cyhalothrin and Chlorfenapyr to N. barkeri

The probit analysis showed clear differences in susceptibility among insecticides and between life stages of *N. barkeri* (Table 2). For adult mites, chlorpyrifos (group A) exhibited the highest toxicity, with LC_10_, LC_20_ and LC_50_ values of 39.42, 68.98, and 130.28 mg/L (designated as A_a_-LC_10_, A_a_-LC_20_ and A_a_-LC_50_), respectively. Chlorfenapyr (group C) showed intermediate toxicity, whereas lambda-cyhalothrin (group B) was the least toxic to adults. A similar pattern was observed for eggs, with LC_50_ values of 159.39 mg/L for chlorpyrifos (A_e_-LC_50_), 222.32 mg/L for chlorfenapyr (C_e_-LC_50_), and 402.87 mg/L for lambda-cyhalothrin (B_e_-LC_50_). In all three insecticides, eggs were less susceptible than adults, as indicated by consistently higher LC_10_, LC_20_, and LC_50_ values.

### 3.3. Sublethal Effects of Egg Exposure on Developmental Duration of the Parental Generation (F_0_)

Exposure of *N. barkeri* eggs to sublethal concentrations affected subsequent developmental duration in a dose-dependent manner (Table 3). In general, LC_10_ treatments caused only slight changes in individual developmental stages and did not alter the total generation time relative to the control. In contrast, LC_20_ treatments prolonged development, particularly in the chlorpyrifos- and chlorfenapyr-treated groups. The larval stage increased from 1.03 d in the control to 1.65 d in A_e_-LC_20_ and 1.63 d in C_e_-LC_20_, while the protonymph stage increased from 1.98 d in the control to 2.53 d and 2.49 d, respectively. Total generation time was significantly extended by all LC_20_ treatments, reaching 10.24 d in A_e_-LC_20_, 9.67 d in B_e_-LC_20_, and 10.38 d in C_e_-LC_20_, compared with 8.52 d in the control. Among all treatments, C_e_-LC_20_ produced the longest total developmental duration.

### 3.4. Sublethal Effects of Adult Exposure on Life-Table Parameters

**Figure 2:**
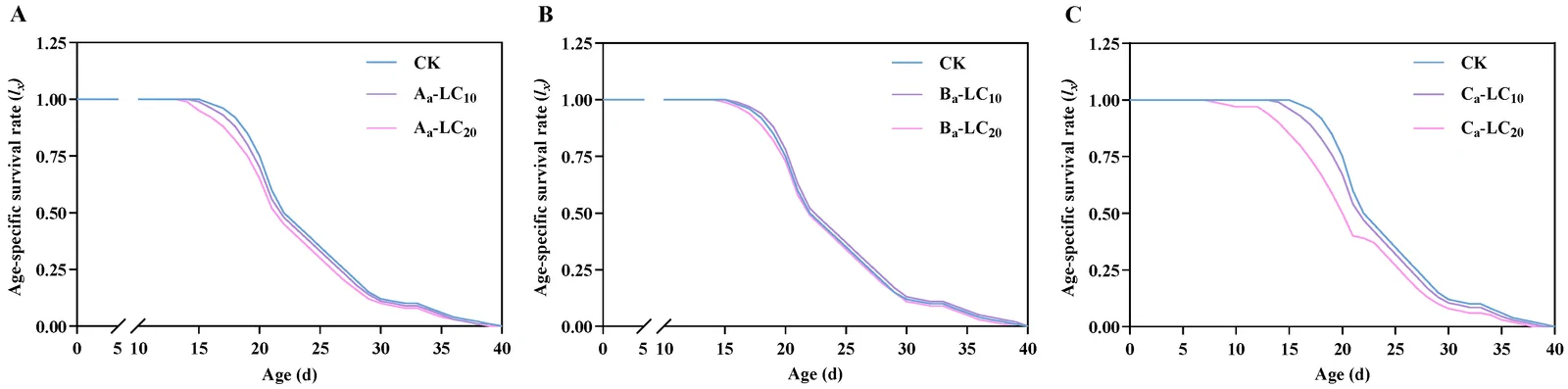
Age-specific survival rate (*l_x_*) of female *Neoseiulus barkeri* over 40 days under control and sublethal pesticide treatments. (**A**) Survival curves for mites exposed to control (CK), A_a_-LC_10_ and A_a_-LC_20_ (chlorpyrifos); (**B**) Survival curves for mites exposed to control (CK), B_a_-LC_10_ and B_a_-LC_20_ (lambda-cyhalothrin); (**C**) Survival curves for mites exposed to control (CK), C_a_-LC_10_ and C_a_-LC_20_ (chlorfenapyr). A_a_-LC_10_, A_a_-LC_20_, B_a_-LC_10_, B_a_-LC_20_, C_a_-LC_10_ and C_a_-LC_20_ represent sublethal concentrations of chlorpyrifos, lambda-cyhalothrin, and chlorfenapyr applied to virgin adult *Neoseiulus barkeri*, respectively.

**Figure 3:**
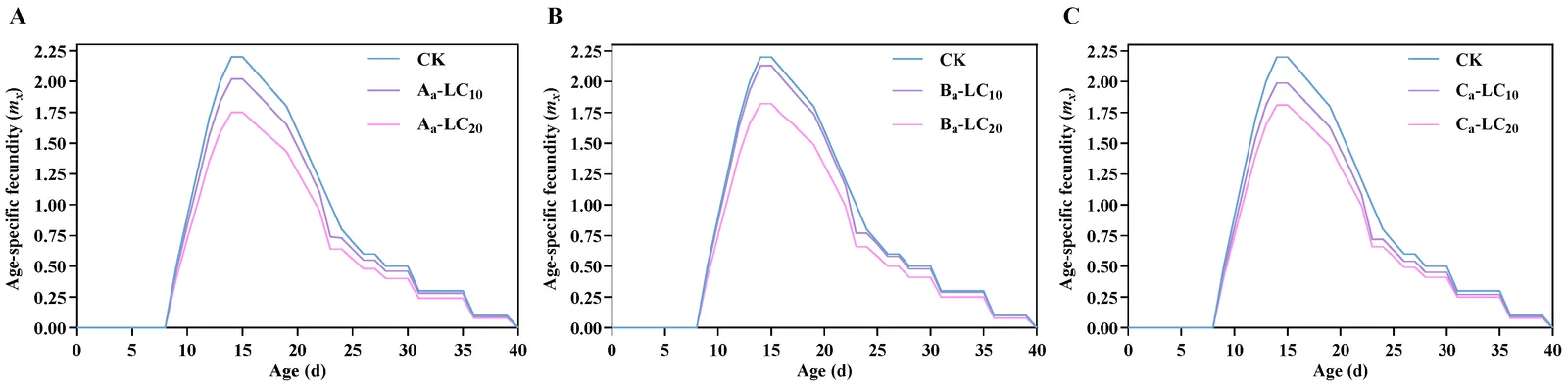
Age-specific fecundity rate (*m_x_*) of female *Neoseiulus barkeri* over 40 days under control and sublethal pesticide treatments. (**A**) Fecundity curves for mites exposed to control (CK), A_a_-LC_10_ and A_a_-LC_20_ (chlorpyrifos); (**B**) Fecundity curves for mites exposed to control (CK), B_a_-LC_10_ and B_a_-LC_20_ (lambda-cyhalothrin); (**C**) Fecundity curves for mites exposed to control (CK), C_a_-LC_10_ and C_a_-LC_20_ (chlorfenapyr). A_a_-LC_10_, A_a_-LC_20_, B_a_-LC_10_, B_a_-LC_20_, C_a_-LC_10_ and C_a_-LC_20_ represent sublethal concentrations of chlorpyrifos, lambda-cyhalothrin, and chlorfenapyr applied to virgin adult *Neoseiulus barkeri*, respectively.

Sublethal exposure at the adult stage reduced the population growth potential of the treated cohort (Table 4). The age-specific survival rate (*l_x_*) and age-specific fecundity rate (*m_x_*) of adult female *N. barkeri* following sublethal pesticide exposure are presented in Figure 2 and Figure 3, respectively. Compared with the control, the LC_20_ treatments decreased the intrinsic rate of increase (*r_m_*) and net reproductive rate (*R*_0_), whereas the population doubling time (*D_t_*) increased. The strongest suppression was observed in Ca-LC_20_, where r_m_ and R_0_ declined to 0.1764 and 15.3122, respectively, and (*D_t_*) increased to 3.9294 d. Aa-LC_20_ also markedly reduced population growth capacity, with r_m_ of 0.1797, and *R*_0_ of 16.6663. By contrast, Ba-LC_10_ remained closest to the control in all life-table parameters. Although mean generation time (*T*) exhibited minor variation across treatments, population doubling time (*D_t_*) increased markedly under LC_20_ exposures, indicating retarded population expansion rather than prolonged individual developmental duration alone.

### 3.5. Sublethal Effects of Adult Exposure on Fecundity and Longevity of F_0_ Females

Direct exposure of virgin adult females to sublethal concentrations also resulted in concentration-dependent reductions in fecundity and longevity (Table 5). Control females laid 35.34 eggs per female and lived for 17.69 d. Chlorpyrifos at LC_10_ significantly reduced female longevity to 15.05 d, although fecundity was not significantly affected. At LC_20_, chlorpyrifos showed the strongest effect, reducing fecundity to 29.03 eggs per female and longevity to 12.39 d. Lambda-cyhalothrin and chlorfenapyr at LC_10_ had little or no significant effect on fecundity, and chlorfenapyr LC_10_ also had no significant effect on adult longevity. However, LC_20_ of both insecticides significantly decreased fecundity and shortened adult lifespan. Overall, chlorpyrifos caused the most pronounced reduction in reproductive performance of F_0_ females.

### 3.6. Transgenerational Effects on the F_1_ Generation After F_0_ Adult Exposure

Maternal exposure of F_0_ adult females to sublethal insecticide concentrations influenced the development of the F_1_ generation (Table 6). LC_10_ treatments produced only minor changes in offspring developmental duration. Under LC_20_ treatments, most developmental stages were only slightly prolonged, but the total generation time of the F_1_ cohort tended to increase. The longest generation time was recorded in C_a_-LC_20_ (9.53 d), which was significantly longer than that of the control (8.71 d). A_a_-LC_20_ and B_a_-LC_20_ also increased total generation time to 9.23 d and 9.25 d, respectively, although these values were not statistically separated from the control. These results indicate that maternal exposure can generate carry-over effects in the next generation, particularly under chlorfenapyr at LC_20_.

## 4. Discussion

This study developed a “four-well volcanic dish” device for toxicity assessment of *N. barkeri* and used it to systematically evaluate the acute contact toxicity of 33 pesticides commonly used in citrus production in China. It also showed that sublethal exposure to chlorpyrifos, lambda-cyhalothrin, and chlorfenapyr at LC_10_ and LC_20_ affects the development and reproduction of *N. barkeri*. Together, these findings provide an important toxicological basis for pesticide selection and for the coordinated use of chemical and biological control in citrus IPM programs [26].

Our results revealed that pesticide toxicity to *N. barkeri* was strongly influenced by pesticide category and mode of action [27]. Amitraz exhibited the highest acute toxicity to *N. barkeri* among all tested compounds. As a non-systemic broad-spectrum formamidine acaricide, amitraz primarily targets octopamine receptors in the arthropod nervous system. Due to the high conservation of octopamine receptors across acarine taxa, amitraz exerts potent toxicity to both phytophagous pest mites and predatory mites, and also shows excellent lethal activity against various arthropod pests including aphids, psyllids and ticks [28].

In contrast, bifenazate and fenbutatin oxide showed extremely low toxicity to *N. barkeri*, indicating high selectivity toward this predatory mite. Bifenazate is a selective contact acaricide. Its main acaricidal activity arises from the inhibition of mitochondrial electron transport chain complex III (Qo site of cytochrome *b*), which blocks cellular respiration and ATP production and results in energy metabolism failure in mites. Xu et al. demonstrated that variations in key cytochrome *b* (cytb) amino acid residues underlie the differential binding affinity of bifenazate to cytb proteins from phytophagous and predatory mites. Bifenazate exhibits >280-fold higher toxicity to phytophagous mites than to predatory mites, indicating strong interspecific selectivity [29]. Fenbutatin oxide is an organotin contact acaricide that exerts toxic effects by inhibiting mitochondrial oxidative phosphorylation and disrupting ATP synthesis. Its extremely low toxicity to *N. barkeri* conforms to the universal pattern observed across the Phytoseiidae family. For instance, the phytoseiid mite *Iphiseiodes zuluagai* exhibits 32.84-fold higher tolerance to fenbutatin oxide than its prey *Oligonychus ilicis* [30]. Multiple independent studies have consistently verified that fenbutatin oxide poses low or negligible risk to commercially mass-reared phytoseiid predators including *Phytoseiulus persimilis* (*P. persimilis*) and *Neoseiulus californicus* (*N. californicus*) [31,32], rendering it an acaricide with good compatibility with biological control. Both bifenazate and fenbutatin oxide display strong selective low toxicity toward phytoseiid mites; their shared core mechanism is interspecific amino acid sequence divergence at the key ligand-binding sites of target proteins between phytophagous pest mites and predatory mites, which drastically reduces the binding affinity between each compound and its target. Similarly, the growth regulators spirodiclofen and pyriproxyfen were also less toxic to adult *N. barkeri*, probably because their primary effects are exerted on immature stages rather than on adults [33]. These selective acaricides may therefore be more compatible with *N. barkeri* release in citrus orchards.

Insecticides posed the greatest non-target risk to *N. barkeri* in this study. Broad-spectrum insecticides, including the organophosphate chlorpyrifos and the pyrethroids lambda-cyhalothrin and beta-cyfluthrin, showed extremely high acute toxicity, with LC_50_ values far below their recommended field concentrations. This result agrees with the general view that non-selective neurotoxic insecticides are highly disruptive to phytoseiid mites and can seriously interfere with biological control in the field [34]. Therefore, these insecticides should be used with great caution in citrus orchards where *N. barkeri* is released or conserved. If their application is unavoidable, an adequate safety interval should be considered before predator release.

By contrast, selective insecticides with novel modes of action were much safer for *N. barkeri* [35]. In the context of this study, “novel modes of action” describe a new generation of insecticides targeting insect-specific physiological sites, in contrast to conventional broad-spectrum agents such as organophosphates and pyrethroids, which act on evolutionarily conserved nervous system targets shared across most arthropod taxa. This high target specificity translates to markedly lower toxicity to non-target beneficial arthropods.

For example, chlorantraniliprole is a selective diamide insecticide that specifically targets ryanodine receptors (RyRs) unique to lepidopteran and hemipteran insects [36]^.^ However, substantial amino acid sequence divergence exists at the key ligand-binding sites of RyR proteins between phytoseiid mites and insects [37], which prevents this compound from exerting potent toxic effects on predatory mites. Consequently, chlorantraniliprole displays very low toxicity to *N. barkeri*. These compounds have relatively low non-target toxicity to beneficial arthropods and may be more suitable for integration into IPM programs involving *N. barkeri*.

Fungicides and herbicides were generally less toxic to *N. barkeri*. Except for prochloraz and mancozeb, all tested fungicides had LC_50_ values above 20,000 mg a.i./L, indicating low acute toxicity to the predatory mite. Among herbicides, glyphosate showed no acute toxicity, whereas glufosinate-ammonium showed moderate toxicity. This difference may be attributed to their distinct modes of action and contact toxicity: glufosinate-ammonium is a non-selective contact herbicide with a certain contact toxicity to arthropods [38,39]. while glyphosate is a systemic herbicide with extremely low toxicity to non-target arthropods. In practice, direct spraying of glufosinate-ammonium onto the citrus canopy should be avoided to reduce non-target exposure of *N. barkeri*.

According to the pesticide database of the China Pesticide Information Network, the field application concentrations of broad-spectrum pesticides commonly used in Chinese citrus orchards mostly fall within the range of 50–2000 mg a.i./L. Pesticides with LC_50_ values of 100–1000 mg a.i./L have median lethal concentrations in the same order of magnitude as recommended field rates. After spraying, residues undergo natural degradation and rain dilution, sustaining sublethal concentrations such as LC_10_ and LC_20_ for extended periods, representing a realistic exposure scenario for predatory mites in the field. In this study, six pesticides had LC_50_ values ranging from 0 to 1000 mg a.i./L, namely amitraz, chlorpyrifos, chlorfenapyr, beta-cyfluthrin, lambda-cyhalothrin and glufosinate-ammonium (Table 1). Among them, amitraz exhibited an extremely low LC_50_, and field-recommended application doses led to nearly complete acute mortality of *N. barkeri*, making persistent sublethal colonies unavailable and invalidating field-oriented sublethal risk analysis. Beta-cyfluthrin shares an identical mode of action with lambda-cyhalothrin within the pyrethroid group, so lambda-cyhalothrin was retained as the representative pyrethroid insecticide to avoid repetitive mechanistic testing. As an herbicide, glufosinate-ammonium is exclusively applied for inter-row weed control rather than canopy spraying in citrus orchards, resulting in rare direct exposure of predatory mites and limited ecological non-target risks compared with foliar insecticides and acaricides.

In contrast, chlorpyrifos, lambda-cyhalothrin and chlorfenapyr belong to organophosphate, pyrethroid and pyrrole groups with distinct toxicological modes of action, which are dominant foliar pesticides for citrus pest management. Their LC_50_ values are comparable to field application rates, and LC_10_ and LC_20_ sublethal concentrations are readily generated during pesticide residue degradation. These three compounds were therefore suitable for characterizing long-term adverse effects of disparate toxic pathways on the development, reproduction and transgenerational fitness of predatory mites, supporting targeted ecological risk assessment for integrated pest management programs. The present study further demonstrated that even sublethal exposure to these insecticides adversely affected the development, reproduction, and population growth of *N. barkeri*, with clear concentration-dependent effects and transgenerational impacts.

Based on LC values, chlorpyrifos was the most toxic compound to both adults and eggs, whereas lambda-cyhalothrin showed the lowest direct toxicity. However, when sublethal effects were considered, chlorfenapyr, especially at LC_20_, caused the strongest demographic suppression after egg exposure, as indicated by the lowest *r_m_*, *λ*, and *R*_0_, and the highest *D_t_*. The life-table results further showed that sublethal exposure at the egg stage can impair the long-term population performance of *N. barkeri*. Reductions in *r_m_*, *λ*, and *R*_0_ indicate slower population growth and lower reproductive capacity, whereas a longer *D_t_* indicates delayed population recovery [40]. Among the three insecticides, chlorfenapyr and chlorpyrifos imposed stronger latent fitness costs than lambda-cyhalothrin. In particular, C_e_-LC_20_ and A_e_-LC_20_ markedly reduced population growth potential. This means that even when individuals survive initial exposure, their later contribution to predator population increase may be greatly reduced. From an IPM perspective, this is important because effective biological control depends not only on survival, but also on rapid reproduction and sustained population buildup.

Direct exposure of adult females produced a somewhat different pattern. Chlorpyrifos caused the greatest reduction in female longevity and fecundity, indicating strong impairment of survival and reproduction in the treated generation. By contrast, chlorfenapyr at LC_10_ had little detectable effect on either trait, suggesting a weaker short-term effect on adult females at low concentration. However, chlorfenapyr at LC_20_ still reduced both fecundity and longevity and produced the clearest transgenerational effect in the F_1_ generation. This stage-dependent discrepancy in sublethal impact is consistent with previous findings across multiple phytoseiid species. For chlorfenapyr, a mitochondrial respiration inhibitor, egg-stage exposure consistently causes stronger suppression of population growth than adult exposure in both *N. californicus* and *P. persimilis*, which is attributed to the high energy demand of embryonic development that makes eggs more vulnerable to respiratory disruption [41]. Similarly, lambda-cyhalothrin exhibits lower acute toxicity to adult phytoseiids but significantly reduces egg hatchability, a pattern linked to differential expression of voltage-gated sodium channel targets across developmental stages [42]. For chlorpyrifos, the stronger acute lethality in adults aligns with its neurotoxic mode of action targeting acetylcholinesterase, whereas egg exposure tends to induce latent fitness costs that manifest in subsequent developmental stages [43,44].

The F_1_ results further indicate that maternal exposure can generate carry-over effects across generations. Although most LC_20_ treatments caused only moderate increases in offspring developmental duration, chlorfenapyr at LC_20_ significantly prolonged the total generation time of the F_1_ cohort. This suggests that the negative effects of insecticide exposure may persist beyond the directly treated generation. Such transgenerational fitness reduction is not unique to *N. barkeri*, but has been widely documented in other phytoseiid mites and arthropod natural enemies. In *Neoseiulus longispinosus*, maternal sublethal acaricide exposure consistently prolongs offspring development and reduces fecundity, driven by reduced yolk protein allocation, insufficient embryonic energy reserves, and transgenerational epigenetic regulation of detoxification enzymes [45]. A cross-taxa comparison further indicates that mitochondrial respiration inhibitors like chlorfenapyr generally induce stronger transgenerational effects than neurotoxic insecticides such as chlorpyrifos and pyrethroids, as energy deficiency can be directly transmitted to offspring via maternal egg provisioning [46]. This mechanistic explanation aligns well with our observation that chlorfenapyr LC_20_ caused the most prominent delay in F_1_ generation time.

Notably, similar pesticide-specific patterns of lethal and sublethal effects have been observed in other key biological control agents, including the predatory ladybeetle *Harmonia axyridis* and the green lacewing *Chrysoperla carnea* [47,48]. The consistency across taxonomically diverse natural enemy taxa indicates that the differential toxicity profiles are intrinsically determined by the mode of action of each insecticide, rather than being species-specific artifacts. This further reinforces that risk assessments relying solely on adult acute mortality will systematically underestimate the long-term impacts of pesticides on natural enemy populations, and highlights the need to incorporate egg-stage exposure and transgenerational endpoints into standard ecotoxicological evaluation frameworks.

Overall, all three insecticides imposed measurable sublethal effects on *N. barkeri*, especially at LC_20_. Chlorpyrifos showed the highest acute toxicity to both adults and eggs and caused the strongest reduction in female longevity and fecundity, whereas chlorfenapyr caused the greatest demographic costs after egg exposure and the clearest transgenerational effect in the F_1_ generation. By contrast, lambda-cyhalothrin showed relatively weaker sublethal effects on population performance. These findings indicate that pesticide compatibility with *N. barkeri* should be evaluated using both lethal and sublethal endpoints. From an IPM perspective, chlorpyrifos and chlorfenapyr should be used with particular caution in citrus orchards where *N. barkeri* is released or conserved.

## Figures and Tables

**Table 1:** Toxicity of 33 tested pesticides to *Neoseiulus barkeri*.

Types	Substances	Slope ± SE	LC_50_ (mg ai/L)	95% CI	χ^2^
Acaricides	Amitraz (EC)	1.79 ± 0.12	55.92	42.52–73.52	7.40
	Pyridaben (EC)	1.98 ± 0.17	8754.58	7292.62–10,509.63	1.29
	Propargite (EW)	1.64 ± 0.04	1235.65	961.03–1588.74	10.00
	Spirodiclofen (SC)	1.31 ± 0.05	3622.63	2780.94–4719.07	4.51
	Azocyclotin (SC)	1.39 ± 0.11	8048.20	5635.04–11,494.78	7.93
	Fenpyroximate (SC)	1.47 ± 0.09	1460.21	852.32–2114.18	22.10
	Bifenazate (SC)	/	>40,000	/	/
	Fenbutatin Oxide (SC)	/	>20,000	/	/
	Tolfenpyrad (SC)	1.43 ± 0.20	2452.92	1969.45–3055.08	8.74
Insecticides	Chlorpyrifos (EC)	2.45 ± 0.24	130.28	112.9–150.32	12.37
	Chlorfenapyr (SC)	1.98 ± 0.13	184.03	124.74–231.25	20.13
	Beta-cyfluthrin (EW)	1.87 ± 0.22	324.90	271.03–389.52	26.84
	Lambda-cyhalothrin (EC)	3.01 ± 0.19	232.96	188.07–297.21	5.26
	Flupyradifurone (SL)	2.47 ± 0.09	4488.59	4208.32–4789.63	8.26
	Thiamethoxam (WDG)	/	>40,000	/	/
	Imidacloprid (SL)	1.30 ± 0.08	14,335.89	8801.05–23,351.48	2.89
	Nitenpyram (AS)	2.38 ± 0.23	9672.35	8310.44–11,257.45	13.02
	Pyriproxyfen (EC)	3.56 ± 0.31	3604.60	2985.46–4097.56	25.32
	Abamectin (EC)	1.55 ± 0.14	2064.83	1693.98–2516.87	11.15
	Buprofezin (WP)	/	>20,000	/	/
	Spirotetramat (SC)	/	>40,000	/	/
	Chlorantraniliprole (SC)	2.07 ± 0.32	7554.99	5768.72–10,337.64	1.54
Fungicides	Mancozeb (WP)	1.56 ± 0.11	7564.73	5959.11–9602.95	12.03
	Fluazinam (SC)	/	>40,000	/	/
	Captan (WDG)	/	>20,000	/	/
	Pyraclostrobin (SC)	/	>20,000	/	/
	Prochloraz (EC)	1.52 ± 0.18	4686.95	3759.63–5842.99	10.21
	Copper Hydroxide (WP)	/	>20,000	/	/
	Thiophanate-Methyl (WP)	/	>20,000	/	/
	Difenoconazole (WDG)	/	>20,000	/	/
	*Bacillus subtilis* (WP)	/	>20,000	/	/
Herbicides	Glufosinate-ammonium (AS)	1.78 ± 0.26	238.57	195.11–291.72	7.69
	Glyphosate (AS)	/	>40,000	/	/

**Table 2:** Sublethal concentrations of chlorpyrifos, lambda-cyhalothrin and chlorfenapyr on adult mites and eggs of *Neoseiulus barkeri*.

Group	Insecticide	Mite State	Slope ± SE	LC_10_ (mg ai/L)	LC_20_ (mg ai/L)	LC_50_ (mg ai/L)
A	Chlorpyrifos	Adult	2.45 ± 0.24	39.42	68.98	130.28
		Egg	2.28 ± 0.11	42.89	77.84	159.39
B	Lambda-cyhalothrin	Adult	1.87 ± 0.22	61.13	110.58	324.90
		Egg	2.03 ± 0.30	80.69	166.47	402.87
C	Chlorfenapyr	Adult	1.98 ± 0.13	41.07	77.43	184.03
		Egg	2.56 ± 0.32	49.96	95.08	222.32

**Table 3:** Developmental duration of *Neoseiulus barkeri* following egg exposure to sublethal concentrations of chlorpyrifos, lambda-cyhalothrin and chlorfenapyr.

Treatment	n/Three Repetitions	Egg Period (d)	Larval Stage (d)	Protonymph Stage (d)	Postnymph Stage (d)	Preoviposition Period (d)	Generation (d)
CK	57	1.87 ± 0.13 ab	1.03 ± 0.04 c	1.98 ± 0.11 b	1.75 ± 0.12 ab	1.88 ± 0.09 ab	8.52 ± 0.25 c
A_e_-LC_10_	52	1.96 ± 0.05 a	1.21 ± 0.10 bc	1.92 ± 0.06 b	1.84 ± 0.03 ab	1.81 ± 0.11 b	8.75 ± 0.22 c
A_e_-LC_20_	39	2.03 ± 0.17 a	1.65 ± 0.08 a	2.53 ± 0.12 a	1.98 ± 0.11 ab	2.05 ± 0.09 a	10.24 ± 0.41 ab
B_e_-LC_10_	55	1.81 ± 0.03 b	1.17 ± 0.05 bc	2.02 ± 0.15 b	1.71 ± 0.18 b	1.93 ± 0.25 ab	8.64 ± 0.47 c
B_e_-LC_20_	47	1.93 ± 0.12 ab	1.44 ± 0.20 b	2.31 ± 0.24 ab	1.86 ± 0.16 ab	2.12 ± 0.20 a	9.67 ± 0.36 b
C_e_-LC_10_	53	1.98 ± 0.09 a	1.08 ± 0.04 bc	2.05 ± 0.19 b	1.67 ± 0.20 b	1.84 ± 0.07 ab	8.62 ± 0.54 c
C_e_-LC_20_	42	2.11 ± 0.18 a	1.63 ±0.11 a	2.49 ± 0.23 a	2.04 ± 0.06 a	2.10 ± 0.12 a	10.38 ± 0.32 a

Note: A_e_-LC_10_, A_e_-LC_20_, B_e_-LC_10_, B_e_-LC_20_, C_e_-LC_10_ and C_e_-LC_20_ represent the sublethal doses of chlorpyrifos, lambda-cyhalothrin, and chlorfenapyr on the eggs of *Neoseiulus barkeri*, respectively. Different lowercase letters indicate significant differences among different treatments at the same developmental stage at the 0.05 level.

**Table 4:** Life-table parameters of F_0_ generation of *Neoseiulus barkeri* following exposure to sublethal concentrations of chlorpyrifos, lambda-cyhalothrin and chlorfenapyr.

Treatment	Intrinsic Rate of Increase (*r_m_*)	Finite Rate of Increase (*λ*)	Net Reproductive Rate (*R_0_*)	Mean Generation Time (*T*)	Population Doubling Time (*D_t_*)
CK	0.1975 ± 0.0074 a	1.2183 ± 0.0122 a	22.4210 ± 1.0231 a	15.7463 ± 1.0211 a	3.5095 ± 0.1656 b
A_a_-LC_10_	0.1909 ± 0.0033 a	1.2103 ± 0.0549 a	19.9978 ± 0.9547 b	15.6933 ± 0.9698 a	3.6312 ± 0.2748 ab
A_a_-LC_20_	0.1797 ± 0.0085 b	1.1968 ± 0.0341 a	16.6663 ± 0.9896 c	15.6582 ± 1.1738 a	3.8578 ± 0.2003 a
B_a_-LC_10_	0.1954 ± 0.0049 a	1.2158 ± 0.0487 a	21.9136 ± 1.0031 a	15.7999 ± 1.0907 a	3.5475 ± 0.1024 b
B_a_-LC_20_	0.1841 ± 0.0044 b	1.2021 ± 0.0223 a	18.2296 ± 1.1003 bc	15.7674 ± 0.9675 a	3.7647 ± 0.1051 a
C_a_-LC_10_	0.1888 ± 0.0057 ab	1.2079 ± 0.0265 a	19.2036 ± 1.1124 b	15.6482 ± 1.4021 a	3.6704 ± 0.2387 ab
C_a_-LC_20_	0.1764 ± 0.0037 b	1.1929 ± 0.0364 a	15.3122 ± 1.2031 c	15.4684 ± 1.2034 a	3.9294 ± 0.2004 a

Note: A_a_-LC_10_, A_a_-LC_20_, B_a_-LC_10_, B_a_-LC_20_, C_a_-LC_10_ and C_a_-LC_20_ represent the sublethal doses of chlorpyrifos, lambda-cyhalothrin, and chlorfenapyr on the virgin adult of *Neoseiulus barkeri*, respectively. Mean generation time (*T*) refers to the average time interval between the birth of maternal individuals and the production of their offspring. Population doubling time (*D_t_*) represents the time required for total population size to double, which is calculated from the intrinsic rate of increase (*r_m_*). Different lowercase letters indicate significant differences among different treatments at the same developmental stage at the 0.05 level.

**Table 5:** Longevity and fecundity of F_0_ female *Neoseiulus barkeri* following exposure to sublethal concentrations of chlorpyrifos, lambda-cyhalothrin and chlorfenapyr.

Treatment	n/Three Repetitions	Number of Eggs/Female	Female Adult Longevity (d)
CK	24	35.34± 2.11 a	17.69 ± 1.13 a
A_a_-LC_10_	24	33.05 ± 1.06 ab	15.05 ± 1.01 b
A_a_-LC_20_	24	29.03 ± 1.23 c	12.39 ± 1.24 c
B_a_-LC_10_	24	34.05 ± 1.44 a	15.47 ± 0.98 ab
B_a_-LC_20_	24	31.69 ± 2.07 bc	13.66 ± 1.33 bc
C_a_-LC_10_	24	35.58 ± 1.64 a	16.01 ±1.04 a
C_a_-LC_20_	24	32.08 ± 1.06 b	13.58 ± 1.30 bc

Note: A_a_-LC_10_, A_a_-LC_20_, B_a_-LC_10_, B_a_-LC_20_, C_a_-LC_10_ and C_a_-LC_20_ represent the sublethal doses of chlorpyrifos, lambda-cyhalothrin, and chlorfenapyr on the virgin adult of *Neoseiulus barkeri*, respectively. Different lowercase letters indicate significant differences among different treatments at the same developmental stage at the 0.05 level.

**Table 6:** Developmental duration of F_1_-generation *Neoseiulus barkeri* following F_0_ females exposure to sublethal concentrations of chlorpyrifos, lambda-cyhalothrin and chlorfenapyr.

Treatment	n/Three Repetitions	Egg Period (d)	Larval Stage (d)	Protonymph Stage (d)	Postnymph Stage (d)	Preoviposition Period (d)	Generation (d)
CK	36	1.92 ± 0.08 ab	1.54 ± 0.06 a	1.81 ± 0.15 a	1.66 ± 0.07 a	1.75 ± 0.11 a	8.71 ± 0.33 b
A_a_-LC_10_	33	2.02 ± 0.13 ab	1.48 ± 0.12 a	1.85 ± 0.11 a	1.63 ± 0.10 a	1.83 ± 0.35 a	8.80 ± 0.18 b
A_a_-LC_20_	37	2.25 ± 0.10 a	1.52 ± 0.23 a	1.93 ± 0.09 a	1.77 ± 0.24 a	1.76 ± 0.18 a	9.23 ± 0.29 ab
B_a_-LC_10_	35	1.88 ± 0.14 b	1.58 ± 0.20 a	1.90 ± 0.24 a	1.70 ± 0.13 a	1.73 ± 0.20 a	8.81 ± 0.15 b
B_a_-LC_20_	32	2.10 ± 0.07 a	1.61 ± 0.32 a	1.98 ± 0.27 a	1.74 ± 0.28 a	1.81 ± 0.14 a	9.25 ± 0.22 ab
C_a_-LC_10_	29	2.07 ± 0.16 a	1.43 ± 0.04 a	1.82 ± 0.07 a	1.69 ± 0.13 a	1.74 ± 0.12 a	8.77 ± 0.30 b
C_a_-LC_20_	35	2.14 ± 0.11 a	1.59 ± 0.11 a	2.11 ± 0.31 a	1.80 ± 0.29 a	1.88 ± 0.23 a	9.53 ± 0.24 a

Note: A_a_-LC_10_, A_a_-LC_20_, B_a_-LC_10_, B_a_-LC_20_, C_a_-LC_10_ and C_a_-LC_20_ represent the sublethal doses of chlorpyrifos, lambda-cyhalothrin, and chlorfenapyr on the virgin adult of *Neoseiulus barkeri*, respectively. Different lowercase letters indicate significant differences among different treatments at the same developmental stage at the 0.05 level.

## Data Availability

The original contributions presented in this study are included in the article. Further inquiries can be directed to the corresponding authors.
